# The Evaluation of Time Performance in the Emergency Response Center to Provide Pre-Hospital Emergency Services in Kermanshah

**DOI:** 10.5539/gjhs.v7n1p274

**Published:** 2014-09-28

**Authors:** Mohsen Mohammadi, Ashkan Nasiripour Amir, Mahmood Fakhri, Ahad Bakhtiari, Samad Azari, Arash Akbarzadeh, Ali Goli, Mohammad Mahboubi

**Affiliations:** 1 Student Research Committee, Kermanshah University of Medical Sciences, Kermanshah, Iran; 2 Department of Health Services Management, School of Management and Economics, Science and Research Branch, Islamic Azad University, Tehran, Iran; 3 Faculty of Paramedical, Kermanshah University of Medical Sciences, Kermanshah, Iran; 4 Tehran University of Medical Sciences, Tehran, Iran; 5 Health Management and Economics Research Center, Iran University of Medical Sciences, Tehran, Iran; 6 School of Publice Health, Tehran University of Medical Sciences, Tehran, Iran; 7 Kermanshah University of Medical Sciences, Kermanshah, Iran; 8 Abadan School of Medical Sciences, Abadan, Iran

**Keywords:** performance, prehospital emergency, emergency medical services, response time

## Abstract

This study evaluated the time performance in the emergency response center to provide pre-hospital emergency services in Kermanshah. This study was a descriptive retrospective cross-sectional study. In this study 500 cases of patients from Shahrivar (September) 2012 to the end of Shahrivar (September) 2013 were selected and studied by the non-probability quota method. The measuring tool included a preset cases record sheet and sampling method was completing the cases record sheet by referring to the patients’ cases. Data were analyzed using SPSS version 18 and the concepts of descriptive and inferential statistics (Kruskal-Wallis test, benchmark Eta (Eta), Games-Howell post hoc test). The results showed that the interval mean between receiving the mission to reaching the scene, between reaching the scene to moving from the scene, and between moving from the scene to a health center was 7.28, 16.73 and 7.28 minutes. The overall mean of time performance from the scene to the health center was 11.34 minutes. Any intervention in order to speed up service delivery, reduce response times, ambulance equipment and facilities required for accuracy, validity and reliability of the data recorded in the emergency dispatch department, Continuing Education of ambulance staffs, the use of manpower with higher specialize levels such as nurses, supply the job satisfaction, and increase the coordination with other departments that are somehow involved in this process can provide the ground for reducing the loss and disability resulting from traffic accidents.

## 1. Introduction

Each year more than 5 million deaths and over 100 million disabilities occur due to injuries happen due to violence, road traffic accidents, falls, burns and drowning (Report, 2003). The goal of emergency response center is to provide medical care to those in need (Arreola et al., 2000). In other words, emergency medical services respond the specific health needs of people outside the hospital. These needs include paying attention to life-threatening injuries, transfer of patients and injuries to the care centers and moving them between centers and mission readiness in the event of health risks but are not limited to these (Barnett et al., 2006). Most people in the world lack access to formal pre-hospital emergency care (Brice et al., 2000). Emergency response center should be simple, consistent and efficient (Charles, 2003). There are two types of response from pre-hospital emergency systems in the different countries; dispatching ambulance with advanced equipment regardless of the type of event immediately after receiving the first call; and receiving the information from the caller, collecting and classifying them and selecting the type and level of service dispatched to the scene (Charles, 2003, Patrick et al., 2005, Charles, 2004). In Iran, the emergency 115 that provides the pre-hospital services was launched in 1976, following the collapse of the roof of Mehr Abad airport terminal (Bidari and et al., 1386). Also, according to the World Health Organization in this country at every 24 hours 102 people and per year about t 37 thousand people lose their lives in accidents (Gasb, 2008). Average growth rate of pre-hospital emergency missions is more than 16% and so approximately every six years, the number of pre-hospital emergency missions will double (Ebarhimian et al., 1391). On the other hand, 50 percent of deaths occur in road accidents in the first hour and 25 percent of the deaths occur during the transfer to the hospital (Jalali, 1380). The results of a case study about emergency patients indicate that the most errors in preventable cases included delays in primary care, lack of adequate care in patient transport and improper communication (Siddiqui et al., 2004). The study of Nasiri pour et al. (2009) evaluated the performance of emergency in Iran as relatively good (Nasiripur et al., 2010), and According to the Accounting Regulations of s comprehensive coverage of pre-hospital emergency services in 2007 by the Council of Ministers to enhance the performance indicators for pre-hospital emergency care, The researchers were to investigate the time performance of Emergency Response Center in providing the pre-hospital emergency services in the city of Kermanshah in 2013.

## 2. Methods

The study is cross - sectional. The population included all the people who were transferred to medical centers with the help of Emergency Response Center in Kermanshah and had completed the emergency care report form. The study was carried out on missions done from the early (September) 2012 to the end of (September) 2013. To determine the sample size, firstly all the missions that their documentation was recorded in emergency disaster management center in Kermanshah were counted (2800 cases), and then due to the statistical population 338 samples should have been examined. All specifications of people that were approved in the report form of emergency 115 by the Ministry of Health and Medical Education (general characteristics of the patient, time record, ambulance personnel code, ambulance kilometers, type of emergency, symptoms, early detection of illness, causes of accidents, medication history, medical history, vital signs, lung, trauma, treatment, medical advice, mission results and innocence letter) were set by a researcher-made check and data were recorded precisely. Reporting forms that were incomplete or had no signature and date were excluded. Sampling method was Non-probability quota and based on the ten urban areas of Kermanshah as 10 percent of the target population. Data were analyzed using SPSS version 18 and the concepts of descriptive and inferential statistics of Kruskal-Wallis test, benchmark Eta (Eta), Games-Howell post hoc test, Spearman correlation coefficients and U post hoc test.

## 3. Result

Results showed that out of a total of 500 missions, the gender of 35.1% of patients in the emergency missions were female and 64.9% were male. About the number of patients sent to hospitals, the maximum number was related to Taleghani Hospital with a 38.3% (191 cases) and the minimum number was related to Dr. Mohamed kermanshahi Hospital with 5.2% (26 cases) and the distribution of events in terms of the reason of patient contact was accidents (21.6% and seizure 4.4 percent) were the highest and the lowest (Table 1).

**Table 1:** Distributing emergency missions due to the type of accident

Type of accident	Accident	Psychological Factors	Fall	Poisoning	Cardiovascular and respiratory disease	Pain (abdomen, side and head)	Loss of consciousness	Trauma	Hysteria	Others	Total
Frequency	108	24	43	63	63	49	43	58	22	26	500
Frequency percent	21.6	4.8	8.6	12.6	12.6	9.8	8.6	11.6	4.4	5.2	100

Results showed that the maximum time spent rescuing people is related to the interval between reaching to the scene to moving from the scene and then is related to the interval between moving from the scene to reaching the hospital. The interval between receiving the mission to reaching the scene (T1) in 500 emergency missions is 7.28 minutes and the interval between reaching to the scene to moving from the scene is 16.73 minutes. The interval between moving from the scene to reaching the hospital is 12.72 minutes and the interval mean between moving from the scene to reaching the hospital is 11.34 minutes. (Table 2).

**Table 2:** Statistical indicators related to the Emergency performance

Variable	frequency	extent	Standard deviation	Minimum	Maximum
(T1) Response Time	500	7	3.81	1	31
(T2) Scene Time	500	13	8.35	0	55
(T3) Transport Time	500	10	7.02	1	40
(T4)	500	8	6.44	0	40

In order to compare the time average of relief efforts in various groups of events Kruskal-Wallis analysis was used. The relationship between the type of time and accident related to the emergency measures (T2) was obtained using standard Eta (Eta) equal to 37/1% showing a moderate correlation between the two variables. Mann-Whitney Post hoc U test showed that the traffic group was significantly different from falling traffic, poisoning, allergies, cardiovascular and respiratory diseases, pain, loss of consciousness, seizures, and trauma related to the emergency procedures at the mean error of 0/001 (*P* – *Value* < 0.001).

Correlation between the type of accident and complete delivery of the patient to the hospital (T4) was obtained using the standard Eta (Eta) as 22.5% showing a relatively weak relationship between the two variables. Games-Howell post hoc test revealed that has a significant difference with “intoxication and Sensibility” and “Traffic” with loss of consciousness” in the mean time of complete delivery of the patient to the hospital.(*P* – *Value* < 0.05).

To investigate the association between work shift and complete delivery of the patient to the hospital (T4), the relationship between these two variables was obtained as 15.6% using the benchmark Eta (Eta) showing a weak relationship between these two variables. Mann-Whitney Post hoc U test showed that only group “20-14” and “8-20” were significantly different with each other in the mean time to complete the delivery of the patient to the hospital (*P* – *Value* < 0.016).

A weak and inverse relationship exists between the area where the accident occurred and the amount of time spent in the full delivery of patient to the hospital. Mann-Whitney Post hoc U test showed that the areas of 1 has a significant difference with the areas of 6 and 8, area of 6 with the areas of 5 and 7, area of 7 with the area 8, at the average time related to the full delivery of the patient to a hospital at the 0/001 error level (*P* – *Value* < 0.001).

To investigate the relationship between the hospital and the full delivery of the patient to a hospital (T4) using the standard Eta (Eta, the relationship between these two variables obtained as 21.5% showing a relatively modest relationship between the two variables. Games-Howell test results showed that Taleghani hospital had a significant difference with Farabi Hospital, Imam Khomeini Hospital and Kermanshahi Hospital at 5% error level at the average time related to the full delivery of the patient to a hospital. (Table 3), (*P* – *Value* < 0.05).

**Table 3:** Indicators of Kruskal-Wallis test

	Chi square	Degrees of freedom	P-Value
Time of relief efforts	82.36	9	*0.00
The relationship (or differences) between the type of accident and the full delivery of the patient to a hospital	27.17	9	*0.04
The relationship (or differences) between the work shift and the full delivery of the patient to a hospital	9.62	2	*0.00
The relationship (or differences) between the area and the full delivery of the patient to a hospital	35.65	9	*0.00
The relationship (or differences) between the hospital and the full delivery of the patient to a hospital	16.12	5	*0.00

## 4. Discussion

Given the importance of the issue that pre-hospital emergency is Confluence with the health sector of the community and the management of reaching to the patient is as an important factor in evaluating the performance of EMS in standard protocol this time should be less than 8 minutes (Peleq & Pliskin, 2004) in the pre-hospital emergency care, In the present study, The interval between receiving the mission to reaching the scene (T1) in 500 emergency missions is 7/28 minutes and The Campbell study (Jack et al., 2007) in 2007, with an average response time over the standard time 9/8 and Klindirer’s study (Kleindorfer et al., 2003) In 2003, emergency missions (97-93%) with an average response time of 10 minutes Study of Jack Campbell and Tomony Gridly (Campbell & Gridley, 2008) in 2008, 1059 mission in 1945 mission review, with an average response time 2/8 minutes and the study of Panahi and colleagues (Panahi et al., 2006) have an average of 15.10 minutes at Tehran show and study (2008), 39.6% of the missions over standard time (over 8 hours) due to traffic has been noted (Shabghare & Dehghanian, 1387), is inconsistent with the findings of this study and higher than the standard level (less than 8 minutes) are This can be due to less time to send the closest ambulance to the location in the city of Kermanshah mission, within the limits of the city of Kermanshah, the proper functioning of the personnel, the appropriate number of ambulances, he said. Accordingly strategic alternatives ambulances and ambulance emergency services due to the occurrence of congestion areas Also calls from an area, resulting in decreased dispatched from bases other than base and thus reduce the response time (Polland et al., 2006).

Rakei and Naderi (2000) in their study was the effect of the vehicle (ambulance) and the distance between the areas of accountability over the world have pointed out (Rakei & Nader, 2002). The report of the Commission is also Farzad Panahi Health Assembly in 2008 due to the geographical distribution and emergency response time refers to the number of bases, Databases so that increasing the number of ambulances, emergency can in the shortest possible time is (Peyravi & Tubaei, 2009). Jarl et al. (2007) in their study pointed to the importance of the distribution of ambulances and poor management of the distribution in the different regions have been effective in the long-term response (Jarrell et al., 2007).

The gender of the patients requesting ambulance of the emergency center, revealed that the 64.9% male, and 35.1% female, with studies Thanyan and colleagues (Tahanian et al., 1385) and glory (Jalali, 1380) greater number of men than women reported consistent Yes, it would be because more men work outside the home, is a type of job and career events. Of the patient with an emergency, accident (21.6%) were the most frequent cause seizures (4.4 percent), with findings of Thanyan et al (Tahanian et al., 1385), consistent with the study Panahi(Panahi et al., 2006) and study in Moradian Fars Province (Moradian, 1383) and also a study (Jalali, 1380) in Tehran.

Indicators are important when assessing the health of the EMS system and, in general, the time, as one of the most important issues related to emergency services are listed in (Altintas & Bilir, 2001) and the effectiveness of services provided, that is the range ten is a good time (Breen et al., 2000). The overall performance of the ambulance service in the first two performance measures (the waiting time of patients) T1 and (when serving patients) T2 is identified. Accurate record of time in the care of patients with critical conditions is needed for legal purposes. In our country, as in many countries, little information exists in the field of pre-hospital emergency services (Altintas & Bilir, 2001). Note that the index of the emergency, as one of the most important factors is the quality of pre-hospital emergency (Peleg & Pliskin, 2004).

The results of this study indicate that the emergency system requires some modifications to achieve a level of international standards. Ambulance personnel performance improvement (Al-Ghamdi, 2002), increasing the number of ambulances (Fischer et al., 2000; Altintas & Bilir, 2001), working to get other forces such as fire and police personnel in some of the events (Jermyn, 2009; Smith et al., 2001) and placing applicants vital equipment in the nearest possible distance (Fedoruk et al., 2002), an emphasis on public education and specific diagnosis and earlier intervention, unit dispatch with the help of trained personnel, quality improvement, given the necessary advice to the callers are able to do. In addition, the system records the information required urgent dispatch of units with precision, reliability and durability are appropriate. Any intervention to increase or decrease the speed of service response time, emergency equipment and facilities required continuing education staff ambulances, use of manpower nurses with higher, Occupational Increasing coordination satisfaction Section the other kind are involved in this process (police, fire fighter, Red Cross) can reduce the background and provide disability related traffic accidents.

## 5. Conclusion

According to the study, the response time in Kermanshah (7.28 min) standard time (8 minutes) is less than the threshold is relatively good, so you can better manage existing resources and modern equipment, educational planning for personnel and the general public and increase public participation, a more accurate assessment of the number of ambulances and emergency equipment and facilities according to population density and also requested the people in each region, is planning to promote timely and appropriate use of emergency culture, Appropriate intersectional coordination between devices such as public utilities, traffic monitoring on the part of health care delivery system, to further improve the pre-hospital emergency system helping to increase.

## References

[ref1] Al-Ghamdi A (2002). Emergency medical service rescue times in Riyadh. Accid Anal Prev.

[ref2] Altintas K, Bilir N (2001). Ambulance times of Ankara emergency aid and rescue services’ ambulance system. Eur J Energ Med.

[ref3] Arreola C, Mock C, Wheatly L. L (2000). Low-costimprovement in prehospital trauma care in a Latin Americancity. J Trauma.

[ref4] Barnett A, Segree W, Matthews A (2006). The Roles and Responsibilities of Physicians in Pre-Hospital Emergency Medical Services:A Caribbean Perspective. West Indian Med J.

[ref5] Bidari A (1386). Performance evaluation of pre-hospital emergency patients transferred to the hospital, the Holy Prophet (SAW). Journal of Tabriz University of Medical Sciences.

[ref6] Breen N, Woods J, Bury G, Murphy A, Brazier H (2000). A national census of ambulance response times to emergency calls in Ireland. J Accid Emerg Med.

[ref7] Brice J, Garrison H, Evans A (2000). Study design andoutcomes in out of hospital emergency medicine research:A10-year analysis. Prehosp Emerg Care.

[ref8] Campbell J, Gridley P (2008). Measuring Response Intervals in a System With a 911 Primary and Emergency Medical Services Secondary Public Safety Answering Point. Ann Emerg Med.

[ref9] Charles A (2003). Emergency medicine in South Africa. J Emerg Med.

[ref10] Charles N (2004). International EMS systems:The United States:Past, present and future. Resuscitation.

[ref11] Ebarhimian A, Khalesi N, Mohamadi G, Tourdeh M, Naghipour M (1391). Transportation Management in Pre-hospital Emergency with Physiological Early Warning Scores. Health Management.

[ref12] Fedoruk J, Currie W, Gobet M (2002). Locations of cardiac arrest:affirmation for community Public Access Defibrillation(PAD) Program. Prehospital Disaster Med.

[ref13] Fischer A, O’Halloran P, Littlejohns P, Kennedy A, Butson G (2000). Ambulance economics. J Public Health Med.

[ref14] Gasb J (2008). WHO.

[ref15] Jack P. C, Kathy S. K, Daniel J. L, William A. W (2007). Measuring the Call-Receipt-to- Defibrillation Interval:Evaluation of Prehospital Methods. Ann Emerg Med.

[ref16] Jalali A (1380). Epidemiological study of patients admitted to hospital emergency rooms nationwide in 1378-79 shawl. EMS magazine in the country.

[ref17] Jarrell B, Tadros A, Whiteman C, Crocco T, Davis S (2007). National health line responses to a stroke scenario:Implications for early intervention. Stroke.

[ref18] Jermyn B (2009). Response interval comparison between urban fire departments and ambulance services. Prehosp Emerg Care.

[ref19] Kleindorfer D, Schneider A, Kissela B, Woo D, Khoury J, Alwell K, Pancioli A (2003). The effect of race and gender on patterns of rt-PA use within a population. Journal of Stroke and Cerebrovascular Diseases.

[ref20] Moradian M (1383). Study of 115 Emergency Call 2 months depending on the mission in Shiraz. Quality Control and Emergency Center Emergency Fares magazine.

[ref21] Nasiripur A. A, Bahadori M. K, Tofighi S, Gohari M. R (2010). Prehospital emergency performance in Iran View of comprehensive coverage plan. Critical care nursing.

[ref22] Panahi F, Khatami M, Azizabadi M, Khoddami H. R, Assari S (2006). Time Indices of Pediatric Prehospital Emergency Care in Tehran. J Iran University of Medical Sciences.

[ref23] Patrick W, Harald H, Walter M (2005). International EMS. Aus Resusc.

[ref24] Peleg K, Pliskin J (2004). A geographic information system simulation model of EMS:reducing ambulance responsetime. Am J Emerg Med.

[ref25] Peyravi M, Tubaei F (2009). Pourmohammadi K. The Efficiency of Motorlance in Comparison with Ambulance in Shiraz.

[ref26] Polland N, Beck K, Rhonda J, Stephen R (2006). Intermediate health emergency care and transportation of the sick and injured. MA 01776.

[ref27] Rakei M, Nader F (2002). Mean Arrival Time for Patients with Head Injury in Mobasher Kashani Hospital in 2001. Journal of Shiraz University of Medical Sciences.

[ref28] Report T. W. H (2003). Shaping the future. In:PUBLICATION, W. (ed.).

[ref29] Shabghare M, Dehghanian A (1387). Mean Time from Calling to EMS till Arrival at Hospital and its Causes (PhD Thesis), Shiraz University of Medical Sciences.

[ref30] Siddiqui A, Zafar H, Bashir S (2004). An audit of head trauma care and mortality. J Coll Physicians Surg Pak.

[ref31] Smith K, Peeters A, Mcneil J (2001). Results from the first 12 months of a fire first-responder program in Australia. Resuscitation.

[ref32] Tahanian M, Okhvat R. H, Mehr A. Z (1385). Average time to reach the accident site by pre-hospital personnel in Golestan province during 1385. Journal of Nursing and Midwifery, Gorgan.

